# The safety of catheter ablation for premature ventricular contractions in patients without structural heart disease

**DOI:** 10.1186/s12872-018-0913-2

**Published:** 2018-08-31

**Authors:** Jin-sheng Wang, Yi-gen Shen, Ri-peng Yin, Saroj Thapa, Yang-pei Peng, Kang-ting Ji, Lian-ming Liao, Jia-feng Lin, Yang-jing Xue

**Affiliations:** 1 Department of Cardiology, The Second Affiliated Hospital and Yuying Children’s Hospital of Wenzhou Medical University, Xueyuanxi Road, No 109, Wenzhou, 325000 Zhejiang China; 2 Department of Laboratory Medicine, Union Hospital, Fujian Medical University, Fuzhou, 350122 Fujian China

**Keywords:** Premature ventricular contractions, Catheter ablation, Structural heart diseases

## Abstract

**Background:**

Patients with frequent premature ventricular contractions (PVCs) are often symptomatic. Catheter ablation was usually indicated to eliminate symptoms in patients with PVCs-induced cardiomyopathy. Currently, PVCs-ablation is also applied for patients with PVCs and no structural heart diseases (SHD); however, the safety and efficacy of ablation in these patients remains unclear.

**Methods:**

In this retrospective study, data from patients who underwent ablation for PVCs from January 2010 to December 2016 at our hospital was retrieved. Predictors of complications and acute procedural success were evaluated.

**Results:**

A total of 1231 patients (mean age 47.8 ± 16.8 years, 59% female) were included. The overall complication rate was 2.7%, and the most common complication was hydropericardium. Two ablation-related mortalities occurred. One patient died of coronary artery injury during the procedure and the other died from infectious endocarditis. Location (left ventricle and epicardium) was the main predictor of complications, with right ventricular outflow tract (RVOT) predicting fewer complications. The acute procedural success rate was 94.1% in all patients. The main predictor of acute procedural success was RVOT origin, while an epicardial origin was a predictor of procedural failure.

**Conclusion:**

Locations of left ventricle and epicardium were predictors of procedural complications for patients with PVCs. Therefore, ablation is not recommended in these patients. For other origins of PVCs, particularly RVOT origin, ablation is a safety and effective treatment.

## Background

Premature ventricular contractions (PVCs) are relatively common and can be detected in 75% of healthy persons using 48-h Holter [1].

Several studies [2, 3] have demonstrated that radiofrequency ablation was more efficient than pharmacological therapy for eliminating PVCs. It was reported that the overall complication rate of PVCs ablation varied from 3.1 to 5.2% and complication rate of PVCs ablation in EPI location was higher than other locations [4, 5]. However, due to insufficient patient sample, predictors of PVC ablation were not evaluated in these studies. So that we conducted this study to evaluate predictor of PVC ablation.

In addition, the prognosis of PVCs remains controversial. Several studies have demonstrated an association between frequent PVCs and potentially reversible cardiomyopathy [6–10]. But PVCs may be a consequence of subclinical cardiomyopathy and it is impossible to prospectively determine whether the PVCs or cardiomyopathy is the primary issue in one given patient [9–11]. A recent cohort study reported no adverse cardiac events and no decline in overall left ventricular ejection fraction after a 5.6 years follow-up of 239 patients with frequent PVCs and no SHD [12]. This study demonstrated that most patients with PVCs and no SHD would not develop into cardiomyopathy even if untreated. For these patients who have a favorable prognosis, the risks and benefits of catheter ablation remain to be elucidated. So that we conducted a retrospective study to evaluate the predictors of complication and success in patients treated with PVCs ablation.

## Methods

Data from patients who underwent radiofrequency catheter ablation for PVCs from January 2010 to December 2016 at our hospital were retrieved. Patients with SHD were excluded. SHD was confirmed by echocardiography, exercise stress testing, ECG, cardiac catheterization, or a history of prior infarcts. Demographic and clinical data, including age, sex, ejection fraction, PVCs burden, the origin and number of PVCs/24H, and complications were collected. The study protocol was approved by our institutional review board.

PVCs localization was classified as right ventricular outflow tract (RVOT); right ventricle (RV); left ventricular outflow tract (LVOT); left ventricle (LV); epicardium (EPI); and multiple (MULTI) origin. Acute ablation success was defined as the elimination of targeted PVCs at least 30 min after the last ablation procedure.

### Statistical analysis

Categorical data are presented as percentages, and continuous variables are expressed as the mean ± SD. To compare discrete variables, Fisher’s exact test or the chi-square test was applied. Continuous variables were compared using a two-group Student’s *t*-test. We considered *p*-values ≤0.05 as statistically significant without adjustment for multiple testing. All *p*-values resulted from two-tailed tests.

To evaluate predictors of ablation complications and outcomes, a univariate logistic regression was performed to identify potential predictors. Parameters that were associated with a *p*-value < 0.1 were entered into a multivariate logistic regression analysis to assess whether they were independent predictors. Results were reported as odds ratios (ORs) with 95% confidence intervals (CIs). The statistical analysis was performed using SPSS version 24.0 (IBM Corp., Armonk, NY).

## Results

### Patient characteristics

In total, 1231 patients were included. The mean age was 47.8 ± 16.8 years, and 59% were female. The mean LV ejection fraction was 66.0% ± 4.3%. The mean pre-ablation number of PVCs was 18,908.7 ± 10,610.1/24 h. Compared to patients without complications (Table 1), the mean age of patients suffered from complications was significantly higher (*p* < 0.001) and the rate of hypertension was significantly higher (*p* = 0.003). In addition, a higher number of hospital stays (*p* < 0.001) was observed in patients who suffered from complications.

**Table 1:** Patient Characteristics

Data	Patients free from complications	Patients suffered complications	*p* value
Age	47.6 ± 16.4	57.9 ± 14.2	*p* < 0.001
Female	59%	64%	*p* = 0.847
Hypertension	28%	52%	*p* = 0.003
Diabetes	7%	12%	*p* = 0.461
Renal disease	0.5%	3%	*p* = 0.464
Number of PVCs	18,644.1 ± 10,628.8	24,010.8 ± 11,595.8	*p* = 0.075
Heart rate	72.6 ± 9.6	74.5 ± 8.3	*p* = 0.277
LVEF	66.0 ± 4.3	65.9 ± 5.2	*p* = 0.891
LVESD	29.9 ± 3.9	30.1 ± 4.3	*p* = 0.823
LVEDD	47.1 ± 5.0	47.4 ± 4.9	*p* = 0.805
BMI	22.8 ± 3.9	23.0 ± 3.3	*p* = 0.670
CRP	3.8 ± 2.6	3.6 ± 2.6	*p* = 0.659
Hospital stays	5.2 ± 4.4	11.6 ± 7.0	*p* < 0.001
Expense	25,638.7 ± 43,971.1	40,978.5 ± 26,230.0	*p* = 0.05
N=	1198	33	

All data are presented as mean ± SD or in percent. Expense is measured in RMB. PVCs = premature ventricular complexes. LVEF = left ventricular ejection fraction. LVESD = left ventricular end-systolic dimension. LVEDD = left ventricular end-diastolic dimension. BMI = body mass index. CR*p* = C-Reactive protein. Number of PVCs was determined by Holter before ablation. Significant difference was found in patients suffered complication and patients free from complication for age, hypertension and hospital stays

Most patients had RVOT-origin PVCs (56.9%). These patients were younger (46.9 years vs. 48.9 years, *p* = 0.046) and were more often female (64.6% vs. 51.5%, *p* < 0.001) than patients with other origins. Compared to patients who were free from complications (Table 2), patients who suffered complications were less likely to have an RVOT origin (*p* < 0.001), but more likely to have EPI (*p* = 0.003) and LV (*p* < 0.001) origins.

**Table 2:** Distribution of PVC Location

Data	Patients free from complications	Patients suffered complications	*p* value
RVOT	695(58%)	6(18%)	*p* < 0.001
RV	167(14%)	2(6%)	*p* = 0.298
LVOT	117(10%)	6(18%)	*p* = 0.195
LV	117(10%)	11(33%)	*p* < 0.001
EPI	60(5%)	6(18%)	*p* = 0.003
MULTI	42(4%)	2(6%)	*p* = 0.761
N=	1198	33	

RVOT = right ventricular outflow tract; RV = right ventricle; LVOT = left ventricular outflow tract; LV = left ventricle; EPI = epicardial; MULTI = multiple PVCs. Significant difference were found in patients suffered complication and patients free from complication for distribution of RVOT; LV and EPI location

### Patient symptoms

Patients suffered PVCs had various symptoms before the ablation procedure, including palpitations (65%), chest discomfort (42%), and dizziness (12%). Most symptoms were considered mild. Syncope occurred in only 1.9% of patients. There were no significant difference in symptoms encountered between patients that had complications and those that did not (Table 3).

**Table 3:** Patient Symptoms

Data	Patients free from complications	Patients suffered complications	*p* value
Palpitations	65%	52%	*p* = 0.098
Dizziness	12%	12%	*p* = 1.000
Syncope	2%	0%	*p* = 1.000
Shortness of breath	9%	21%	*p* = 0.043
Chest discomfort	42%	55%	*p* = 0.139
Symptoms> 1 month	72%	70%	*p* = 0.801

Most of patients suffer mild symptoms such as palpitation and chest discomfort. None significant difference was found for symptoms in patients suffered complication and patients free from complication

### Complications

**Fig. 1:**
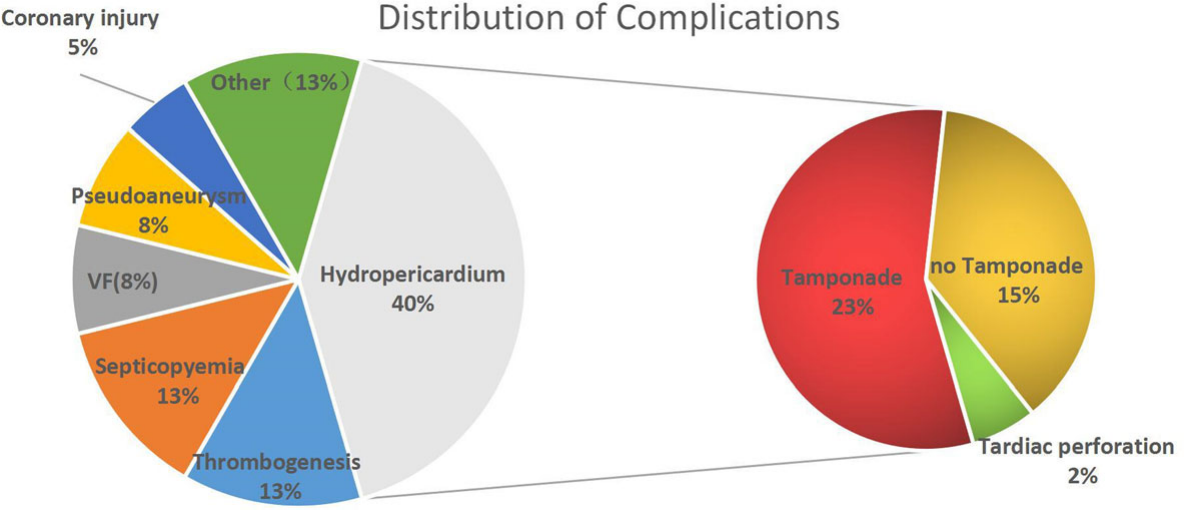
Distribution of complications - The most common complication was hydropericardium. Most of the hydropericardium developed into cardiac tamponade.Two ablation-related mortalities occurred. VF = ventricular fibrillation

In this study, a total of 39 complications occurred in 33 patients (Fig. 1) and the overall complication rate was 2.7%. Major adverse cardiac events (MACE) occurred in 1.5% of patients (acute coronary syndrome, ventricular fibrillation, cardiac tamponade, pulmonary embolism, infectious endocarditis, stroke, and death). Moderate adverse events occurred in 1.2% of patients (pseudoaneurysms, retroperitoneal hematoma, thrombogenesis, septicopyemia, and pericardial effusion). The most common complication was hydropericardium (Fig. 1). Nine patients with hydropericardium developed cardiac tamponade (Fig. 1) and were treated with pericardiopuncture. In addition, two procedure-related deaths occurred in patients with LV and MULTI-origin PVCs. One patient died from injury to the left main coronary artery during ablation, and the other died of infectious endocarditis after ablation. The total mortality rate was 0.16%. Right ventricular perforation happened during ablation in one patient, but this was successfully treated with cardiorrhaphy.

**Fig. 2:**
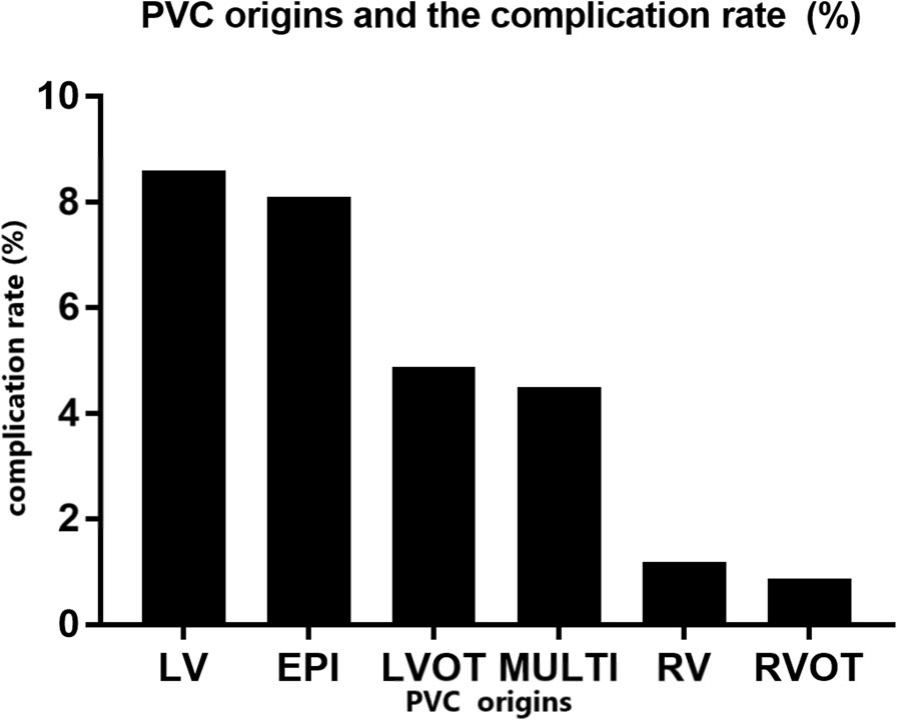
Complication rate and PVC sites - The highest rate of complication was in patients with epicardial origin and the lowest complication rate was in patients with RCOT PVCs

In terms of PVCs origin (Fig. 2), RVOT was associated with the lowest complication rate (0.86%). LV (*p* = 0.001) and EPI origins were associated with the highest complication rate. The majority of procedural complications in patients with LV (53%) and EPI (67%) origins were pericardial effusion.

**Fig. 3:**
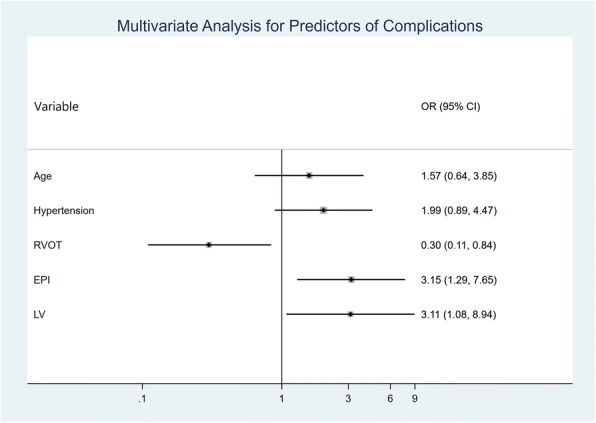
Multivariate analysis for predictors of complications - RVOT location was an independent predictor of procedural success while epicardial location was an independent predictor of procedural failure

As shown in Fig. 3, independent predictors of complications by a multivariate logistic regression analysis included EPI (OR:3.11, 95% CI: 1.08 to 8.94, *p* = 0.04) and LV (OR:3.15, 95% CI:1.29 to 7.65, *p* = 0.01) origins, while RVOT origin (OR: 0.30, 95% CI: 0.11 to 0.84, *p* = 0.02) was a negative predictor of complications.

**Fig. 4:**
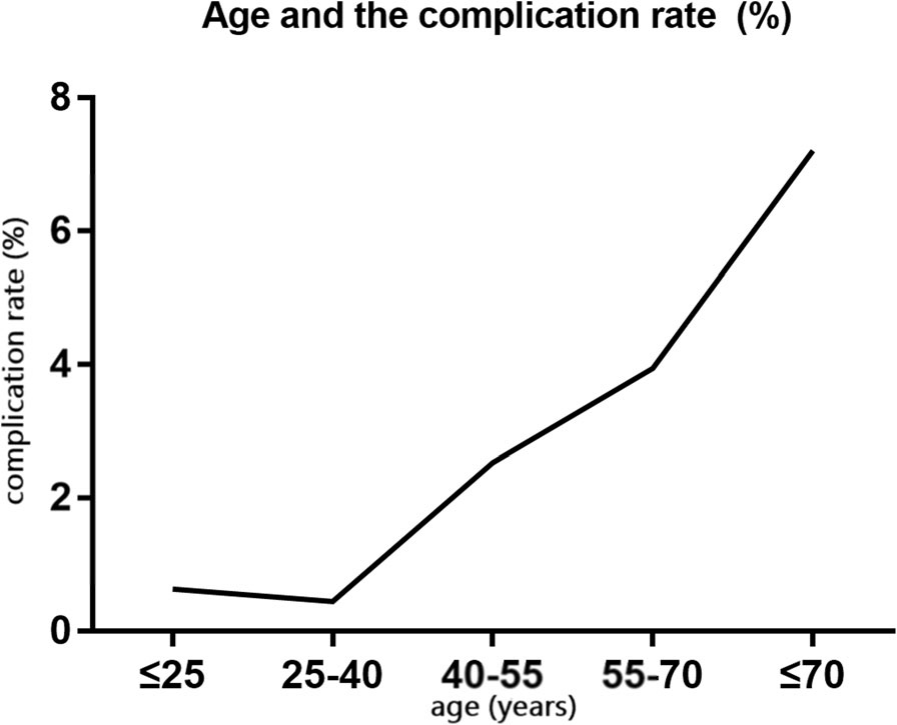
Complication rate and age - complication rate rose roughly positive as age got older

Although age was not an independent predictor of complications, the complication rate tended to increase with age (Fig. 4). Patients older than 70 years had a significantly higher complication rate than those younger patients (*p* = 0.027).

### Procedural outcomes

**Fig. 5:**
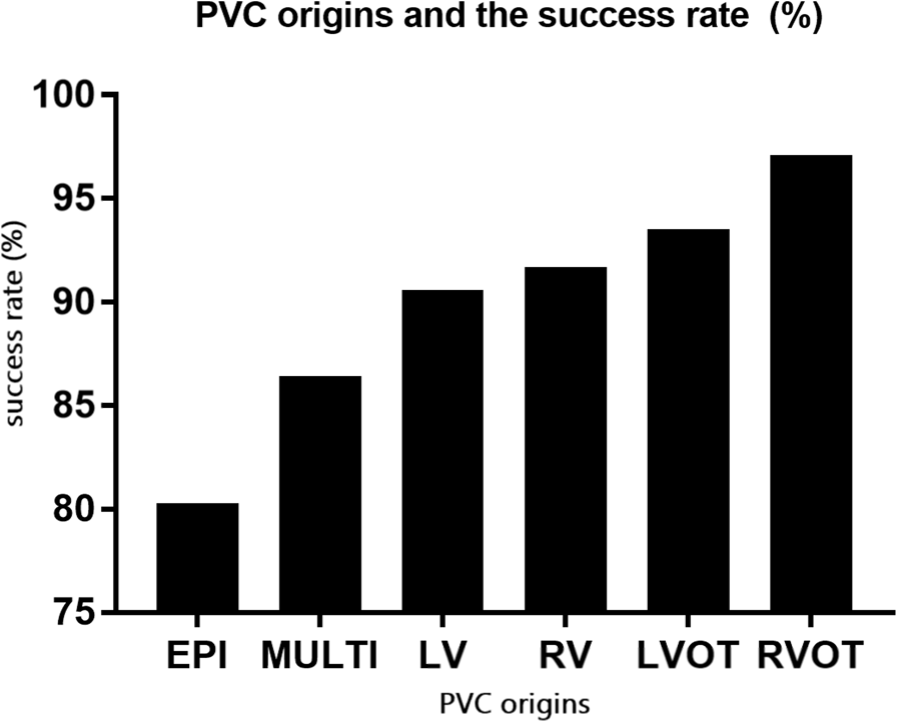
Ablation success and PVC sites - The highest rate of success was in patients with RVOT origin and the lowest success rate was in patients with epicardial PVCs

**Fig. 6:**
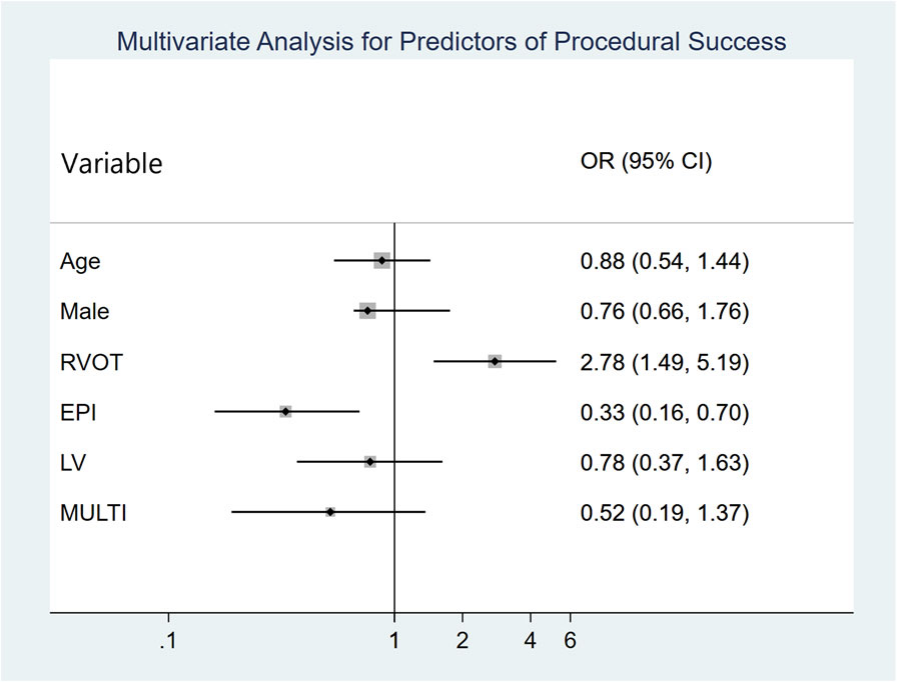
Multivariate analysis for predictors of acute procedural success - Right ventricular outflow tract location and was an independent predictor of procedural success while epicardial location was an independent predictor of procedural failure

Acute procedural success was achieved in 94.1% of all patients (Fig. 5). The highest rate of success was seen in patients with an RVOT origin (97.1%), and the lowest success rate was seen in patients with EPI PVCs (80.3%). A multivariate analysis (Fig. 6) showed that EPI origin was an independent predictor of procedural failure (OR: 0.33, 95% CI: 0.16 to 0.70, *p* = 0.004), while RVOT origin was independently predictive of acute procedural success (OR: 2.78, 95% CI: 1.49 to 5.20, *p* = 0.001).

## Discussion

### Main findings

This is a large sample study that contains a total of 1231 patients who underwent PVCs ablation. The overall success rate was 94.1%. The overall complication rate was 2.7%. By multivariate logistic regression analysis, origins of EPI and LV were independent predictors of complications and RVOT location was the negative predictor of complications. Moreover, RVOT was an independent predictor of the procedural success and EPI was the predictor of procedural failure.

### Safety of PVCs ablation

In this study, a multivariate analysis revealed that LV and EPI origins were independent predictors of the occurrence of complication. Moreover, two procedure-related deaths occurred respectively in patients with LV and MULTI origins. The result in this study demonstrated that PVC ablation in EPI and LV location were under higher operation risk. Given the favorable prognosis of these patients [12], PVC ablation in EPI and LV location is unadvisable. Besides, although age was not an independent predictor of complications in the multivariate analysis, there was a tendency for increased age to be associated with increased complications (Fig. 4). In patients older than 70 years, the expected complication rate was significantly higher before the PVC location was determined. In conclusion, PVCs ablation of patients without SHD should not be recommended when the patients have PVCs of an LV or EPI origin and/or are older than 70 years.

In the 2014 EHRA expert consensus on ventricular arrhythmias [13], catheter ablation for PVCs was recommended for patients who remain symptomatic despite conservative treatment or who have probable reversible LV dysfunction. However, there was no distinction made between the different PVCs sites. This study can provide evidences for the completion of new guidelines.

Previous studies [14–16] reported that different and complex ionic channels alterations and metabolic pro-arrhythmic triggers may lead to ventricular and atrial arrhythmias, conditioning worse prognosis. Celestino Sardu [14, 15] reported that metabolic syndrome (MS) is associated with a poorer outcome in patients affected by PVCs after ablation and MS may affect the functionality of cardiac resynchronization therapy with a defibrillator. In addition, RyR2 channels play an important role in glucose homeostasis and development of cardiovascular disease [16]. However, in our study, body mass index and C-Reactive protein was not significantly higher in patients suffered complications. Reasons may be that patients with SHD, such as coronary heart disease which is more common in metabolic syndrome patients, were excluded in this study and the average body mass index of patients included in our study was obvious lower than the previous researches.

### Efficacy of ablation

The results of this study show that ablation was effective for PVCs with a high overall success rate. Our results are similar to other studies that showed the overall acute success of ablation for PVCs was achieved in 84% and 82% of patients respectively [4, 5] and these studies also showed that acute success was higher in patients with an RVOT origin compared to other origins.

But a multivariate analysis in this study reveals that an EPI origin is predictive of procedural failure, whereas RVOT origin is the only independent predictor of acute successful outcome. In combination with the predictors of complication, obviously, EPI is not a proper operation location while RVOT should be an optimal site. Only patients without SHD were included in our study, which may account for our higher overall success rate.

### Limitations

Patients in this research were from a single center and thus may not be representative of other patient populations. In addition, the operations were performed by experienced surgeons at our center; the outcome may be different for junior surgeons. Furthermore, no data on the number of PVCs or a quantitative assessment of symptom improvement after ablation were available, so that the long-term success was not evaluated.

## Conclusions

For patients without SHD, ablation for PVCs of EPI and LV origins is not recommended due to a remarkably higher complication rate, lower success rate, and risk of mortality. Other origins, particularly RVOT, were optimal operative locations with desirable acute success and complication rate. In addition, age should be considered before the procedure.

## Abbreviations

BMI: Body mass index
CIs: Confidence intervals
CRP: C-Reactive protein
EPI: Epicardium
LV: Left ventricle
LVOT: Left ventricular outflow tract
MULTI: Multiple
ORs: Odds ratios
PVCs: Premature ventricular contractions
RV: Right ventricle
RVOT: Right ventricular outflow tract
SHD: Structural heart diseases
